# Towards Holistic Governance of China’s E-Waste Recycling: Evolution of Networked Policies

**DOI:** 10.3390/ijerph17207407

**Published:** 2020-10-12

**Authors:** Xiuli Yang, Xin Miao, Jinli Wu, Ziwei Duan, Rui Yang, Yanhong Tang

**Affiliations:** 1 School of Public Administration and Law, Northeast Agricultural University, Harbin 150030, China; xiuliyang@neau.edu.cn; 2 School of Management, Harbin Institute of Technology, Harbin 150001, China; miaoxin@hit.edu.cn (X.M.); wisdomhit@126.com (J.W.); 18b910068@stu.hit.edu.cn (R.Y.); 3 School of Humanities, Social Sciences & Law, Harbin Institute of Technology, Harbin 150001, China; 19s116040@stu.hit.edu.cn

**Keywords:** electronic waste (e-waste), recycling, holistic governance, networked policies, government departments

## Abstract

Electronic products are being updated and replaced much faster and there is therefore an increasing growth in electronic waste (e-waste). In order to promote professional recycling of e-waste, the relevant government departments of China have published a series of policies. This paper aims to unearth the evolution tendency of the networked policies towards holistic governance of China’s e-waste recycling. Content analysis, quantitative text analysis and network analysis are applied to analyze relevant policy documents from 2001 to 2016. This paper illustrates evolution of policy themes, evolution of intergovernmental relationships, and evolution of policy relations. This study reveals policy intentions, maps policy progress, and unearths governance philosophy, providing an overall understanding of the policy ways by which the Chinese government has deployed its guiding strategies on professional recycling of e-waste. This paper illustrates how to approach holistic governance from perspective of networked policies, contributing to answering the central question of holistic governance about how to achieve it.

## 1. Introduction

With the rapid development of technology and the popularity of electronic appliances and digital products, the lifecycle time for electronic products has become shorter and shorter, leading to emergence of an immense electronic waste (e-waste) problem. The report of the “Global E-waste Monitor 2017” shows that “in 2016, 44.7 million metric tons of e-waste were generated that is equivalent of almost 4500 Eiffel towers” (see https://www.itu.int/en/ITU-D/Climate-Change/Pages/Global-E-waste-Monitor-2017.aspx). Heavy metal and toxic materials from discharged e-waste can cause serious damage to the environment and public health [1]. On the other hand, e-waste contains an enormous amount of recyclable resources. Adopting sustainable management measures for effective use of e-waste resources plays vital role in reducing the adverse effect of e-waste on the environment and health [2].

Many developed regions such as European Union (EU) and Japan have published relevant laws and policies regarding recycling of e-wastes through motivating producers to design more environmentally-friendly products, which contribute to reducing landfill and reducing the impact on the environment [3]. Mulliner et al. argued that e-waste recycling can effectively reduce cost, landfill land and the impact on the environment [4]. Through summarizing cases from America, Japan and EU about recycling laws, Atasu and Wassenhove concluded that there is a strong need for research on enforcing legislation for operations [5].

The problems of e-waste in emerging economies [6,7,8], such as China [9] and Mexico [10], are prominent. Wealthy countries pursue the maximization of e-waste value in terms of economic, environmental and social benefits, such as creating employment opportunities and meeting the demands of circular economy [11,12,13]. The following aspects have received common attention from emerging economies and wealthy countries.

Firstly, the Extended Producer Responsibility (EPR) [14] is a key point in the field. Legislation based on EPR is popular [15,16]. Gu et al. established a fund balance analysis model on the basis of the Waste Electrical and Electronic Equipment (WEEE) and EPR fund system implemented in China. Cao et al. analyzed the effectiveness of current e-waste management in China from the perspective of government policies, corporations and public awareness, in the context of the EPR-based e-waste management system established in China [15]. Tong et al. proposed a logical basis for planning large collection and sorting centers (equipped with automated equipment) based on the EPR principle, to replace labor-intensive sorting activities that are ubiquitous in “waste villages” [17].

The second one is reverse logistics (RL). Tong et al. used the provincial data of China’s formal e-waste recycling plants in 2014 to quantify the contribution of the informal sectors to e-waste transportation, and recommended optimizing the spatial allocation of recycling capacity based on the dynamics of the recycling market, improving the efficiency of the RL system with innovative business model that includes various stakeholders in the recycling chain [18]. Tong et al. identified three types of e-waste governance: (1) Community programs for all consumers’ waste sorting behavior; (2) RL systems with vending machines connected to traditional business chains; (3) Internet-based solutions for communication between consumers and recyclers [19]. Cao et al. established a WEEE RL system model including individual traders, second-hand stores, retailers using “trade-in” activities, classified recycling stations, informal recycling companies, and professional recycling companies [16]. Isernia et al. provided an illustrative analysis of the distribution of collection centers (CCs) that played a key role in the e-waste collection performance of the Italian provinces and the starting point of the WEEE RL cycle [11].

Thirdly, the concept of stakeholders plays an important role in this field as well. For instance, Wang et al. used a grey decision-making algorithm combined with stakeholder theory to propose a research framework to identify the obstacles faced by network-based e-waste collection systems. Their results demonstrated that the lack of tax incentives and the lack of willingness of low-income people to participate are the two biggest obstacles, suggesting the key points for policymakers to improve Internet-based e-waste collection performance [20]. Tong et al. recommended optimizing the spatial allocation of recycling capacity involving various stakeholders in the recycling chain [18].

Fourthly, the incentive theory has been attached great importance. Xue et al. found that contract incentive mechanisms can increase the enthusiasm of retailers for recycling [21]. Gu et al. established a new mode of operation of the fund to solve the incentive problems of ecological design [22]. Davis and Garb argued for economic incentives to encourage informal recyclers to obtain higher profits from grinding cables rather than burning. At the same time, the top-down regulations (imposing a high fine on the burning cable) can double encourage informal recyclers to grind cables instead of burning them [6].

To sum up, the governance on e-waste are the interaction of multi-stakeholder network. Scholars have noticed the applicability and superiority of networked analysis in studying e-waste recycling. For example, Ingram et al. claimed that networked analysis methods are general and flexible in recognizing and understanding the multiple values of environmental governance [23]. Keskitalo et al. pointed out that social network analysis can provide insight into multiple interaction patterns between organizations and within organizations [24]. Wong drew on the policy network approach to provide an interpretation framework for e-waste governance in Hong Kong and mainland of China, identifying the major barriers to effective cross-border e-waste control and prevention [25]. In view of the superiority of networked analysis methods, it has important application prospects in the research of e-waste governance. Wong also believed that “*institutional arrangements play a dominant role in governing e-waste policy networks at the local level of governance in Hong Kong and China*” [25]. China’s hierarchical bureaucracy structure with fragmented administration authorities make it complicated for governing e-waste recycling. Chinese governmental sectors have released a series of policies over the past years to promote e-waste recycling. Many researches intended to find clues from specific views to study the government role [15,26,27], however, failed to reveal the holistic role of the government.

Holistic governance was originally put forward to make up for the fragmentation of public service provision [28]. It as a theoretical framework is important for understanding the governance on e-waste. It implies a governance philosophy in coping with fragmentation and complexity of policies [29]. Holistic governance often implies the progress of policies and strategies with the advantage of involving local government departments, enterprises and citizens [30,31,32]. A central question for holistic governance is how to make it happen [28]. Most of the previous studies fail to answer this question. To fill this gap, this work illustrates how holistic governance on e-waste is approached from perspective of networked policies.

The aim of this work is to unearth China’s policy progress towards holistic governance on e-waste recycling. Since the existing literature has already discussed many aspects of China’s policies and governance on e-waste recycling [33], to avoid shifting attention and repetitive work, this work did not devote to writing a long literature review on e-waste governance. We limit space to a few previous papers related to the central topic to make the writing succinct and cohesive. The following uses quantitative text analysis to study the research question: How does the Chinese government improve the networked policies to achieve holistic governance of e-waste recycling? This work innovatively considers China’s policy network progress on e-waste recycling as a process towards holistic governance, and explains the roles of the Chinese government departments on promoting e-waste recycling, and empirically answers the central question of holistic governance about the ways in which the organization of holistic governance is achieved by illustrating the evolutionary network effect of China’s e-waste recycling policies.

## 2. China’s Policy System for E-Waste Recycling

Government departments at various levels promote recycling of e-waste through issuing a series of policies. Governments play the role of policy makers and supervisors. The central government put forward macroscopic policy guidance on major aspects of professional recycling of e-wastes. Different national ministries have published a series of measures on professional recycling of e-waste. Among them, the Ministry of Environmental Protection has the most prominent driving force. As the primary responsible department, the Ministry of Environmental Protection has made considerable policies, and used administrative means to supervise workshops and enterprises in dismantling e-waste. General Administration of Customs has played a great role in managing import and export of e-waste. Its strict supervision has helped control the amount to e-waste imported into China. The Ministry of Commerce, Ministry of Industry and Information Technology, Ministry of Finance, and State Administration of Taxation and other ministries have all contributed to making relevant policies.

The Ministry of Environmental Protection is mainly responsible for environment supervision, regulation and production supervision in dismantling and treatment of waste household appliances. This ministry is in charge of qualification censorship and permission on treatment companies of waste electronic product. It also published technical specifications for controlling pollution produced in treatment process of e-waste. It was in charge of environmental investigation on dangerous wastes, providing list on national dangerous wastes and specify types of dangers caused by e-waste. Besides, it also provided education to public and governmental organizations about recycling of e-waste. General Administration of Customs, as the department responsible for supervising imported and exported cargo, mainly manages and supervises the import and export of e-waste. It has published relevant documents restricting e-waste importation and strengthened supervision of e-waste smuggling, especially in the Guangdong region which is the largest importer of e-waste in China. The Ministry of Commerce undertook the responsibility of market regulation of important consumables, circulation management of import production materials and propelling adjustment of the circulation structure. It has established circulation management regulations for e-waste and arranged management of replacing old household appliances with new ones. It is mainly responsible for the supervision and investigation of the professional recycling industry of renewable resources in e-waste and organizing trials of construction of recycling systems for renewable resources. The Ministry of Industry and Information Technology is responsible for drafting and implementing industrial plans, industrial policies and standards to guide the development of the e-waste industry. In professional recycling system of e-waste, the Ministry of Industry and Information Technology is in charge of establishing industrial specifications, such as industrial specifications for the copper smelting industry, promoting industry upgrades, launching green development switch actions, making industrial innovation plans, using science & technology to promote industrial innovation, revising industrial standards, and promoting normalization and standardization of lead acid battery and toxic material, and also propelling pilot EPR of electronic products. The Ministry of Finance and State Administration of Taxation mainly subsidizes energy-saving household appliance production and sale, including special fund subsidies and tax subsidies. The two departments also worked with the Ministry of Environmental Protection, Ministry of Industry and Information Technology, and Development and Reform Committee to publish subsidy standards for e-waste treatment funds and actively promotes the work of replacing old household appliances with new ones.

Local governments are responsible for local recycling of e-waste. Local policies and supervision are more specific and operable. Taking Guangdong Province as an example, the Three-Year Action Plan for Development of New Industry printed and issued by the Guangzhou Municipal Government stipulated that important city minerals such as waste mechanical equipment, cable, waste metal and scrap electronic products have to be recycled, used in scale and high value. The Department of Environmental Protection of Guangdong Province has published notices about professional dismantling and usage of e-waste. Shantou government promoted pilot recycling of old and waste household appliances in Guiling Town, which is a national pilot unit of the circular economy. Provincial governments, municipal environmental protection bureaus, economic and trade committees, municipal governments and other local departments have issued relevant plans and notices to promote professional recycling of e-waste.

Laws, regulations and rules regarding professional recycling of e-waste have different stipulations. Regulations include administrative regulations and local regulations. Administrative regulations are regulations published by the State Council, lower than laws but higher than regulatory documents published by various committees around the country. Compared with laws, stipulations of administrative regulations are more exclusive and specific. Local regulations about relevant standards are established by local governments according to specific local conditions. The rules herein include rules from various departments of the central government and rules published by local governments. The Ministry of Environmental Protection and Ministry of Commerce have published relevant rules on professional recycling of e-waste. Compared with laws and administrative regulations, departmental rules define stipulations on operation specifications and administrative punishments. Some provincial and municipal governments have released local rules on professional recycling of e-waste in local regions.

The government guide and supervise recycling of e-waste in China. Relevant policies including laws, regulations and rules are important tools for driving professional recycling of e-waste. The policy documents have stipulated production behavior, recycling behavior, usage behavior, selling and purchasing behavior of the stakeholders.

## 3. Materials and Methods 

Policy documents have the advantages of wide availability and objectivity [34]. Through content analysis and quantitative text analysis on policy documents, the holistic governance trend of China’s e-waste recycling policies can be unearthed, uncovering the governance philosophy. The use of governmental policy documents is appropriate to examine the variability and variety of e-waste governance landscape, internalizing policy subject, object, goal, and instrument [35].

Content analysis on textual data in political documents can help to mine for evidence of political processes and policy positions [36], and reveal policy evolution and intention [35]. Quantitative text analysis is semantic analysis technique via integration of quantitative and qualitative methods, which enables identification of contextual factors and evolution trend of policies, uncovering the underlying essence and principle [35].

Hidden evidences about governance philosophy can be mined from policy documents. While qualitative text analysis is somewhat subjective, this work employs quantitative text analysis to unearth the governance philosophy by visualizing changes of policy themes and their network characteristics.

This paper conducts a quantitative policy text analysis to capture key features of e-waste recycling policies in different periods [35,37]. Co-word, frequency, and clustering of policy keywords in different stages can reveal policy concerns in different development periods. Co-word analysis, as a technique of content analysis for mining the strength association between themes or ideas in textual data [38], can reflect relations of different policy documents by counting co-occurrence frequencies of keywords in policy documents [37]. Clustering analysis, which is based on the criteria of maximizing the difference between groups and minimizing differences within the group [39], is to gather keywords with close relations to form groups so as to observe hot topics in polices of different periods. In this study, the extracted keyword types are governmental department names, policy document types, important verbs, important approaches, and policy topic words in each policy document. This work confirms keywords according to list of keywords in official documents of the State Council, and establish co-word matrix of high-frequency keywords through word frequency statistics, and then run clustering analysis of keywords to get policy keyword clusters [37] to show which governmental departments enact relevant policy documents, which topics draw more attention from the government, and what are the approaches adopted by the government.

The e-waste recycling related policy documents in 2001–2016 were collected to be the sample of this study for a number of reasons. In 2001, mobile phones began to be prevalent in mainland China; in 2001, the smuggling of e-waste in Guiyu town in Guangdong Province drew wide concern at home and abroad; in 2001, China started the work of restricting harmful substances of electronic products, which is seen as the first step from the government for controlling e-waste; so the year of 2001 is taken as the starting year of the sample analysis. Due to data acquisition limit caused by career change of the well-trained assistants, the data were collected to 2016. The policy documents were collected from governmental official websites and http://www.pkulaw.cn/, which is a policy database established by Law Department of Peking University and is widely used by Chinese scholars in humanities and social sciences. This research adopted the expert discussion method based on content analysis, that is, with the help of well-trained assistants, professionals in this field independently speed-read the content of the collected policies one by one to judge the relevance with e-waste recycling, and then negotiate the different opinions until reaching an agreement [40]. 628 policy documents were thus selected. In order to observe the evolution tendency of China’s e-waste recycling policies and illustrate the evolution difference of the networked policies in different stages, this paper divided the time span into equidistant four stages: 2001–2004, 2005–2008, 2009–2012, and 2013–2016 [35].

## 4. Results

This section consists of three aspects about the evolution of the networked policies, that is, evolution of policy theme networks, evolution of intergovernmental cooperation networks, and evolution of policy relation networks. These can reveal the evolution of China’s e-waste recycling policies and demonstrate the holistic governance intention of the government.

### 4.1. Evolution of Policy Themes

**Figure 1:**
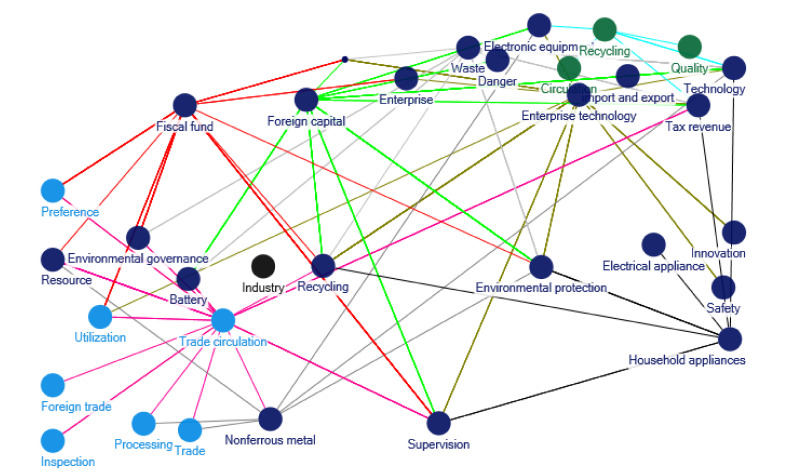
Clustering of policy themes in 2001–2004. Notes: The words in Figure 1 are listed as follows: Fiscal fund; Industry; Preference; Supervision; Enterprise; Environmental protection; Recycling; Environmental governance; Utilization; Resource; Household appliances; Electrical appliance; Technology; Tax revenue; Foreign capital; Import and export; Electronic equipment; Battery; Danger; Waste; Recycling; Quality; Circulation; Trade circulation; Inspection; Foreign trade; Processing; Nonferrous metal; Trade; Enterprise technology; Innovation; Safety.

The starting stage for relevant policy documents regarding e-waste recycling in China occurred during 2001–2004. The clustering of policy themes in 2001–2004 is shown as Figure 1. During this period, for prevention on environmental dangers from e-waste, there are policy documents such as *Announcement of the State Environmental Protection Administration on Strengthening Environmental Management of Waste Electronic and Electrical Equipment,* and *Technical Policy for Pollution Prevention and Control of Waste Batteries*. In this stage, the environmental planning at the national level, such as the *“Tenth Five Year Plan” of National Environmental Protection* and the *“Tenth Five Year Plan” of Energy Conservation and Comprehensive Utilization of Resources* was issued by the State Environmental Protection Administration in 2001, which mentioned the recycling of e-waste.

**Figure 2:**
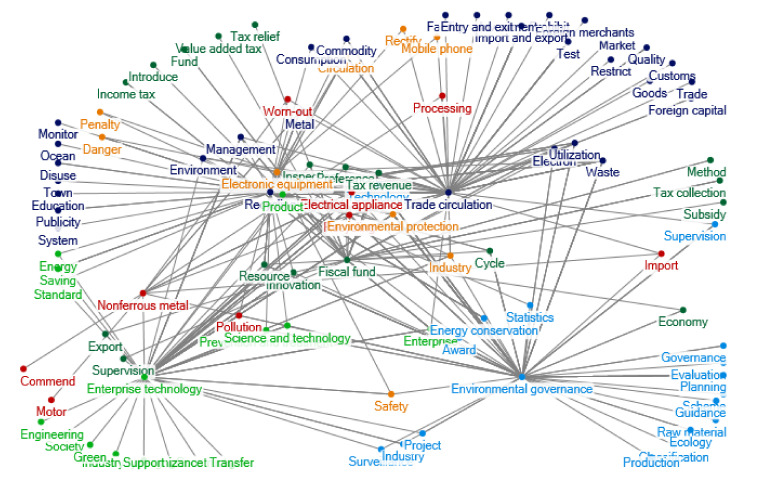
Clustering of policy themes in 2005–2008. Notes: The words in Figure 2 are listed as follows: Fiscal fund; Method; Income tax; Value added tax; Tax relief; Tax collection; Subsidy; Fund; Introduce; Management; Enterprise; Electron; Innovation; Export; Cycle; Utilization; Inspection; Technology; Electrical appliance; Preference; Supervision; Economy; Tax revenue; Resource; Industry; Electronic equipment; Recycling; Circulation; Metal; Rectify; Penalty; Waste; Safety; Environmental protection; Danger; Mobile phone; Environmental governance; Classification; Scheme; Production; Raw material; Guidance; Evaluation; Governance; Planning; Ecology; Supervision; Statistics; Prevention and treatment; Award; Surveillance; Energy conservation; Pollution; Industry; Science and technology; Project; Import; Recycling; Town; Monitor; Education; Ocean; System; Publicity; Disuse; Standard; Consumption; Energy; Environment; Saving; Worn-out; Commodity; Trade circulation; Facility; Market; Goods; Prohibit; Investment; Restrict; Foreign merchants; Customs; Import and export; Test; Quality; Foreign capital; Trade; Entry and exit; Product; Processing; Enterprise technology; Construct; Cognizance; Industry type; Society; Transfer; Support; Green; Engineering; Nonferrous metal; Commend; Motor.

There was a rapid increase in relevant policy documents regarding e-waste recycling in China during 2005–2008. The clustering of policy themes in 2005–2008 is shown as Figure 2. During this period, contents and sources of the policy documents are extended, and there are more new policy themes and keywords. In 2008, the *Circular Economy Promotion Law of the People’s Republic of China* was promulgated and implemented, which regulates the recycling and utilization of e-waste from the perspective of circular economy. The *Management Methods for Recycling of Renewable Resources* covers the recycling management of various resources contained in e-waste. During this period, China’s import and export management of e-waste has also been further clarified, such as the *List of Designated Processing and Utilization Units of Imported Waste Hardwares and Electrical Appliances, Waste Wires and Cables and Waste Motors* issued by the State Environmental Administration in 2007 to restrict small workshops from illegal processing and smelting waste metals.

**Figure 3:**
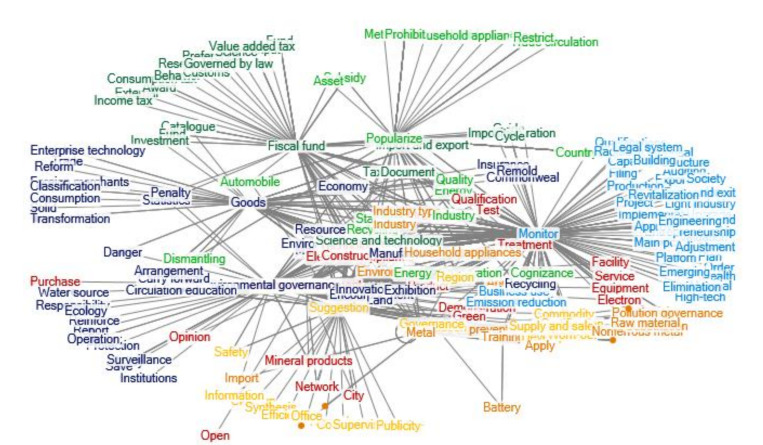
Clustering of policy themes in 2009–2012. Notes: The words in Figure 3 are listed as follows: Fiscal fund; External; Award; Consumption tax; Preference; Fund; Input; Research; Income tax; Behavior; Customs; Science; Value added tax; Governed by law; Catalogue; Management; Enterprise; Tax revenue; Deliberation; Planning; Document; Guide; Work; Imposition; Subsidy; Environmental protection; Statistics; Fund; Electronic Appliance; Investment; Technology; Import and export; Penalty; Standard; Automobile; Recycling; Cycle; Construct; Economy; Disuse; Asset; Science and technology; Industry; Environment; Energy; Industry type; Resource; Electronic equipment; Purchase; Open; Opinion; Service; Equipment; Quality; Facility; Demonstration; Qualification; Electron; Network; Product; Test; Mineral products; Treatment; Appliances; Green; City; Environmental governance; Responsibility; Policy; Report; Institutions; Protection; Operation; Reinforce; Water source; Save; Ecology; Surveillance; Standard of prevention and treatment; Manufacture; Pollution; Training; Encouragement; Governance; Safety; Emission reduction; Energy conservation; Waste; Land; Business use; Danger; Innovation; Import; Recycling; Exhibition; Suggestion; Division of labor; Clear up; Synthesis; Proposal; Complaint; Efficiency; Office; Supervision; Publicity; Information; Cognizance; Dismantling; Commodity; Certification; Development; Circulation education; Worn-out; Region; Industry; Supply and sale; Household appliances; Carry forward; Apply; Arrangement; Popularize; Operation; Method; Brand; To countryside; Trade-in; Household appliances; Trade circulation; Restrict; Prohibit; Countryside; Goods; Trade; Foreign merchants; Solid; Reform; Transformation; Classification; Consumption; Enterprise technology; Commonweal; Insurance; Remold; Monitor; Qualifications; Transfer; Filing; Main point; Platform; Evaluation; Export; Auditing; Implementation; Order; Health; Entrepreneurship; Proposal; Structure; Upgrade; Society; Commend; Capacity; Material; Project; Approve; System; Radiation; Light industry; Emerging; Engineering; Entry and exit; High-tech; Legal system; Elimination; Building; Plan; Adjustment; Production; Revitalization; Supervision; Pollution governance; Nonferrous metal; Substitution; Raw material; Battery; Metal.

There was still growth in the number of relevant policy documents regarding e-waste recycling in China during 2009–2012. The clustering of policy themes in 2009–2012 is shown as Figure 3. During this period, contents and sources of the policy documents are more extensive. The Ministry of Environmental Protection, the Ministry of Commerce, the Ministry of Industry and Information Technology, etc. vigorously pushed the policy of old for new appliances, issuing a number of notices to ensure the smooth development of the replacement of old by new appliances. The Development and Reform Commission and other departments also promoted the policy of old for new cars and proposed transformation and upgrading guidance for the appliance industry. In this period, the “*Measures for the Import Management of Solid Waste* were issued, and the *Catalogue for the Treatment of Waste Electrical and Electronic Products (the First Batch)*” was also issued, and the qualification examination and supplement of waste electrical and electronic products treatment enterprises were audited and managed.

In this stage, the national policies paid more attention to the enterprises of processing and utilization of e-waste, mainly in the form of tax management, technical support, technical innovation commendation and so on. The “*Plan for Adjustment and Revitalization of Nonferrous Metals Industry”* was released. During this period, the recycling policies of e-waste involves a wider scope and specific issue and puts forward specific and operable solutions for a growing number of used household appliances.

**Figure 4:**
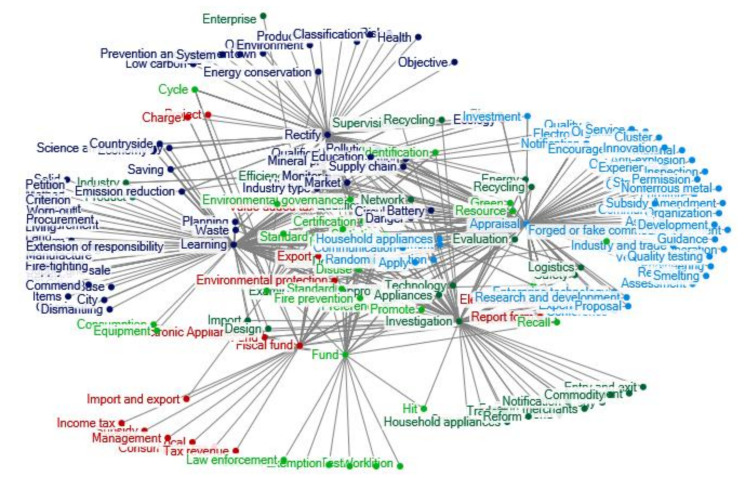
Clustering of policy themes in 2013–2016. Notes: The words in Figure 4 are listed as follows: Fiscal fund; Consumption tax; Subsidy; Local; Tax revenue; Income tax; Management; Catalogue; Fund; Examination and approval; Environmental protection; Standard; Document; Electronic Appliances; Preference Project; Standard; Value added tax; Disuse; Charge; Export; Import and export; Electronic equipment; Report form; Efficiency; Enterprise; Supervision; Governance; Ecology; Industry; Plan; Certification; Qualifications; Quality; Product; Statistics; Safety; Energy conservation; Qualification; Network; Electron; Industry; Logistics; Environmental governance; Notification; Rectify; Cut down; Objective; Scheme; Institutions; Information; Accident; Renewable; Order; Risk; Low carbon; Health; Environment; Production; Prevention and treatment; Classification; System; Green; Utilization; Construction; Investment; Circulation; Monitor; Mineral product; Recycling; Planning; Science and technology; Demonstration; Waste; Danger; Pollution; Evaluation; Saving; Industry type; Cycle; Publicity; Emission reduction; Resource; Energy; Economy; Battery; Market; Countryside Recycling; Identification; Education; Supply chain; Learning; Treatment; Method; Criterion; Base; Clearing; Dismantling; Harm; Land; Benchmarking; Solid; Items; Behavior; Measurement; Waste; Living; Petition; Citizen; City; Supply and sale; Worn-out; Commend; Procurement; Fire-fighting; Manufacture; Extension of responsibility; Development; Promote; Consumption; Random inspection; Communication; Producer; Technology; Import; Appliances; Household appliances; Apply; Fire prevention; Design; Equipment; Fund; Air-condition; Work; Test; Trade circulation; Exemption; Law enforcement; Hit; Recall; Investigation; Prohibit; Trend; Smuggle; Strategy; Entry and exit; Foreign merchants; Regulation; Trade; Adjustment; Notification; Commodity; Household appliances; Reform; Enterprise technology; Conference; Experiment; Research and development; Proposal; Appraisal; Verification; Assessment; Opinion; Organization; Anti-explosion; Experience; Engineering; Furniture; Equipment; Guide; Service; Raw material; Communication; Cluster; Encouragement; Inspection; Operation; Amendment; Forged or fake commodities; Structure; Innovation; Organization; Industry and trade; Region; Quality testing; Asset; Development; Guidance; Nonferrous metal Subsidy; Permission; Smelting.

The number of relevant policy documents regarding e-waste recycling in China during 2013–2016 is still rich and extensive. The clustering of policy themes in 2013–2016 is shown in Figure 4. With the greatly shortened renewal cycle of China’s electronic products and the increase of waste electronic products and household appliances, China’s treatment of e-waste also learned from the experience concerning EPR of foreign producers. For example, the “*Notice on Pilot Work of Extended Producer Responsibility of Electrical and Electronic Products”* from the Ministry of Industry and Information Technology, Ministry of Finance, Ministry of Commerce, etc. The collection system of waste electrical and electronic products treatment fund has become a highlight spot. 

In 2013, the Ministry of Commerce issued measures for the circulation management of used electrical and electronic products. The green lifestyle has also been widely publicized and promoted, and citizens and organizations are encouraged to hand over waste electrical appliances to professional recycling enterprises.

Policy themes of the above four stages show that China’s recycling policies on e-waste have been gradually improved and comprehensive towards holistic governance. The quantity of published policies and related themes basically continues to show an increasing trend. More and more related issues have been taken into account in the policy system.

### 4.2. Evolution of Intergovernmental Cooperation Networks

A policy document is often enacted by multiple government departments. By analyzing the departments involved in cooperation of policy making, the cooperation network map of government departments can be obtained to show the intergovernmental relationships in the progress of forming e-waste recycling policies. The issuing departments of policy documents is deemed as nodes. The node size represents the number of policy documents issued. The larger the node is, the more policies are issued. The thickness of the connection line between two nodes represents the number of cooperative documents issued. The thicker the connecting line is, the more cooperative documents are issued, and the closer the cooperation relationship between the two departments is [35,41].

**Figure 5:**
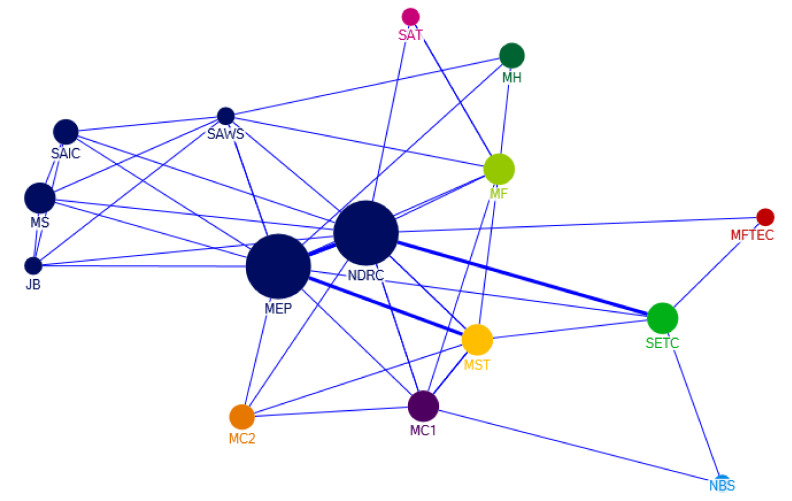
Departmental collaboration network of policy-making on e-waste recycling in 2001–2004. Notes: The abbreviations in Figure 5 are listed as follows; NDRC: National Development and Reform Commission; MC1: Ministry of Commerce; SAT: State Administration of Taxation; MF: Ministry of Finance; MST: Ministry of Science & Technology; MC2: Ministry of Construction; SAWS: State Administration of Work Safety; JB: Judicial Bureau; SAIC: State Administration of Industry and Commerce; MS: Ministry of Supervision; MFTEC: Ministry of Foreign Trade and Economic Cooperation; SETC: State Economy and Trade Commission; MEP: Ministry of Environmental Protection; MH: Ministry of Health; NBS: National Bureau of Statistics.

From 2000 to 2004, some policy documents were issued collaboratively by two or more departments. Figure 5 shows the collaboration network of the policy-making sectors in 2001–2004. At this stage, the Ministry of Environmental Protection and the National Development and Reform Commission are the two major departments in the joint issuance of e-waste recycling policies. In this stage, the cooperative issuance of policies mainly involves the following contents: encouraging the development of industrial policy on professional recycling of e-waste, prevention and control of waste batteries and other e-waste pollution, and smelting standards of e-waste.

**Figure 6:**
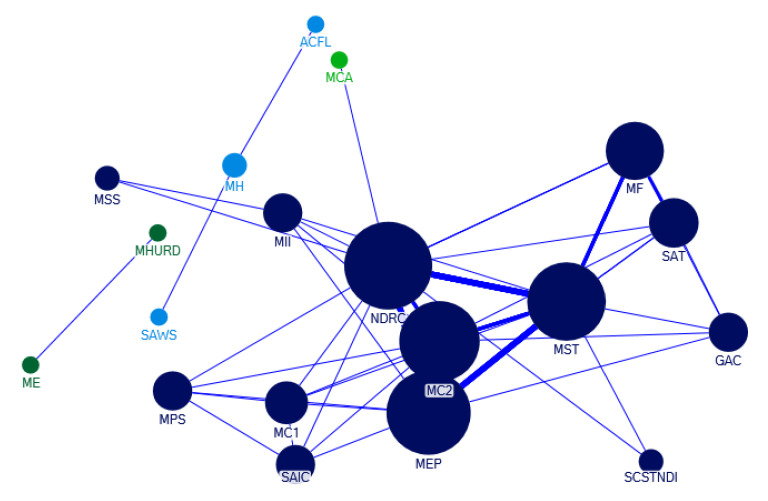
Departmental collaboration network of policy-making on e-waste recycling in 2005–2008. Notes: The abbreviations in Figure 6 are listed as follows: NDRC: National Development and Reform Commission; SCSTNDI: State Commission of Science and Technology for National Defense Industry; MST: Ministry of Science & Technology; MF: Ministry of Finance; SAT: State Administration of Taxation; GAC: General Administration of customs; MCA: Ministry of Civil Affairs; SAIC: State Administration for Industry and Commerce; MEP: Ministry of Environmental Protection; MPS: Ministry of Public Security; MC1: Ministry of Commerce; MC2: Ministry of Construction; MII: Ministry of information industry; MH: Ministry of Health; ACFL: All-China Federation League; SAWS: State Administration of Work Safety; MSS: Ministry of Safety Supervision; MHURD: Ministry of Housing and Urban-Rural Development; ME: Ministry of education.

The quantity of e-waste policy documents released collaboratively increased in 2005–2008. Figure 6 shows the collaboration network of the government sectors for policy-making on e-waste recycling in 2005–2008. During this period, the Ministry of Science and Technology, the National Development and Reform Commission, the Ministry of Environmental Protection and the Ministry of Commerce have more collaborations in policy releases. During this period, the circular economy concept began to be promoted in China, and the country carried out pilot circular economy projects, such as the construction of the e-waste dismantling base in Guiyu Town in Guangdong Province, where manual dismantling workshops were moved to the industrial park, and carried out circular development through technical upgrading and industrial planning. In addition to this policy hot spot, the contents of the joint issuance also include the catalogue of products prohibited from processing trade, governance of electronic product market, pollution prevention and control technology of waste household appliances and electronic products, management system of renewable resources recovery, restrictions on import and export products, etc. The scope of the policies is more extensive, and the governance idea has been advanced compared with the previous stage.

**Figure 7:**
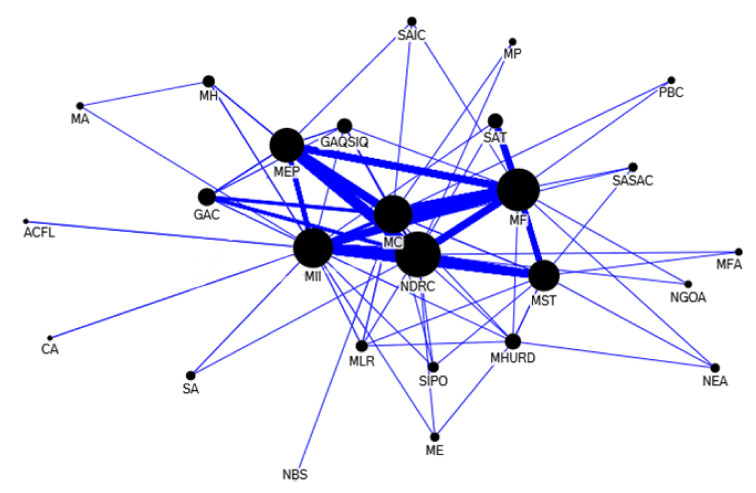
Departmental collaboration network of policy-making on e-waste recycling in 2009–2012. Notes: The abbreviations in Figure 7 are listed as follows: SA: Standardization Administration; GAQSIQ: General Administration of Quality Supervision Inspection and Quarantine; MII: Ministry of information industry; NDRC: National Development and Reform Commission; ACFL: All-China Federation League; NGOA: National Government Offices Administration; MF: Ministry of Finance; SASAC: State-owned Assets Supervision and Administration Commission; MST: Ministry of Science & Technology; MLR: Ministry of Land and Resources; MC: Ministry of Commerce; MHURD: Ministry of Housing and Urban-Rural Development; CA: Certification Administration; NBS: National Bureau of statistics; SIPO: State Intellectual Property Office; PBC: People’s Bank of China; GAC: General Administration of Customs; MEP: Ministry of Environmental Protection; SAIC: General Administration of Industry and Commerce; MH: Ministry of Health; SAT: State Administration of Taxation; NEA: National Energy Bureau; MFA: Ministry of foreign affairs; MA: Ministry of agriculture; ME: Ministry of education; MP: Ministry of propaganda.

In the years of 2009–2012, the number of government departments involved in collaborative release of e-waste policy documents was relatively large, and the main departments are more closely linked. Figure 7 shows the collaboration network of the government sectors for policy-making on e-waste recycling in 2009–2012. During this period, the National Development and Reform Commission, the Ministry of Finance, the Ministry of Environmental Protection, the Ministry of Industry and Information Technology, the Ministry of Commerce, and the Ministry of Science and Technology, have more collaborative policy releases. During this period, the Chinese government vigorously advanced the policy of replacing old appliances with new ones, especially the Ministry of Commerce, the Ministry of Environmental Protection, the Ministry of Finance and other departments jointly issued a number of policy documents on the recycling and replacement of old appliances. The main contents of the collaborative policy documents also involve the following aspects: the management of imported e-waste, the exchange of old and new automotive appliances, technology introduction and innovation, tax preference and subsidies, industrial adjustment and revitalization, industrial development planning, fund system for the treatment of waste electrical and electronic products, professional e-waste recycling publicity, etc. There are some breakthroughs in solving e-waste problems, especially in the implementation of the old for new policies on household appliances, which provides a way for the regular recycling of a large number of used household appliances in China. In addition, the collection of treatment fund for imported household appliances and electronic products has accumulated special funds for the professional dismantling of imported used household appliances in China.

**Figure 8:**
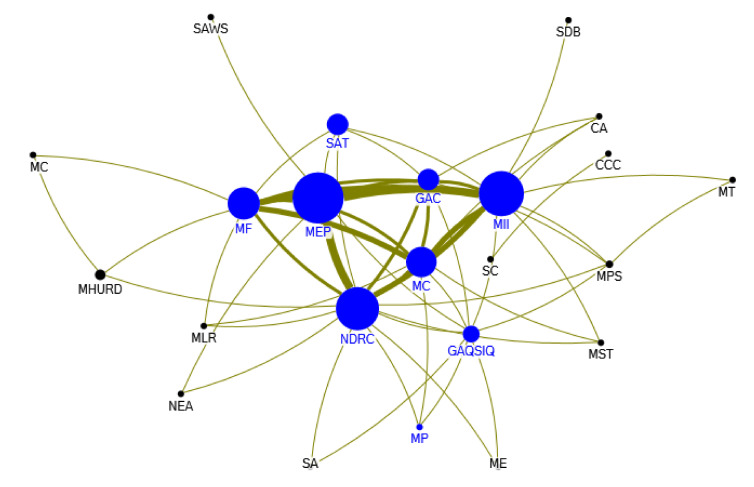
Departmental collaboration network of policy-making on e-waste recycling in 2013–2016. Notes: The abbreviations in Figure 8 are listed as follows: SAT: State Administration of Taxation; MEP: Ministry of Environmental Protection; MII: Ministry of information industry; NDRC: National Development and Reform Commission; GAC: General Administration of Customs; MF: Ministry of Finance; SAWS: State Administration of Work Safety; SC: State Council; CCC: Chinese Central Communist; MST: Ministry of Science & Technology; MC: Ministry of Commerce; MHURD: Ministry of Housing and Urban-Rural Development; MC: Ministry of Culture; SA: Standardization Administration; GAQSIQ: General Administration of Quality Supervision Inspection and Quarantine; MP: Ministry of propaganda; MPS: Ministry of Public Security; ME: Ministry of Education; SDB: State Development Bank; NEA: National Energy Administration; MLR: Ministry of Land and Resources; MT: Ministry of Transport; CA: Certification Administration.

From 2013 to 2016, the Ministry of Environmental Protection, the National Development and Reform Commission, the Ministry of Information Industry, the Ministry of Commerce and the Ministry of Finance are major issuers of collaborative policies on e-waste recycling. Figure 8 shows the collaboration network of the government sectors for policy-making on e-waste recycling in this period. With the increasing concern about environmental issues, the government also paid more and more attention to the environmental pollution caused by e-waste, and actively innovated in policy content by drawing on the advanced experience of e-waste recycling in Japan and Germany. The collaborative policy documents maintained the policy hot spots of the previous stage, including waste electrical and electronic products treatment fund, subsidy system, e-waste import and export management, e-waste standard disassembly guide, and “urban mineral” industrial demonstration park pilot. At this stage, the relatively new policy themes are the construction pilot of ecological civilization industrial park, the pilot of EPR of electrical and electronic products, the professional recycling system of batteries of electric vehicles, etc. With the continuous implementation of the “*Scientific Outlook on Development*”, the relevant government departments pay more and more attention to the recycling of renewable resources, and the professional recycling of electronic products has also been more widely supported. In addition, in view of the environmental hazards caused by e-waste in daily life, the government departments put stress on organization and carrying out environmental protection publicity and education activities.

### 4.3. Evolution of Policy Relations

**Figure 9:**
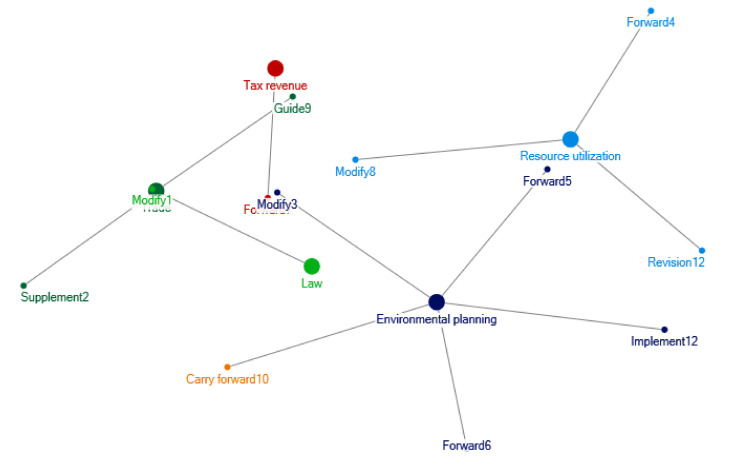
Relations of e-waste recycling policies in 2001–2004.

**Figure 10:**
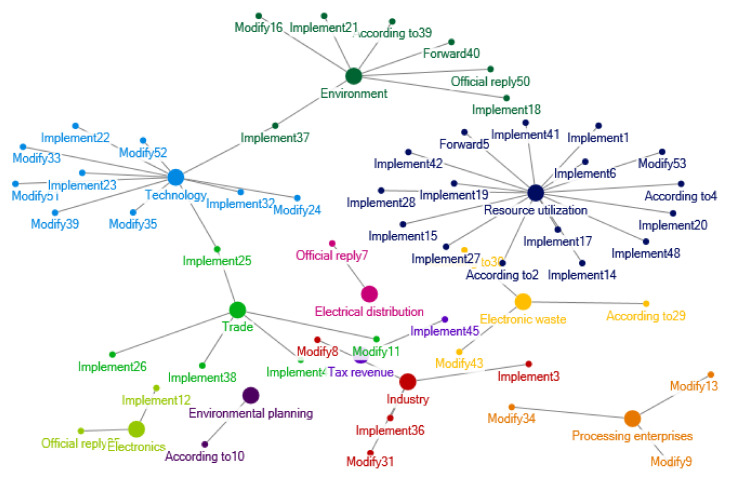
Relations of e-waste recycling policies in 2005–2008.

**Figure 11:**
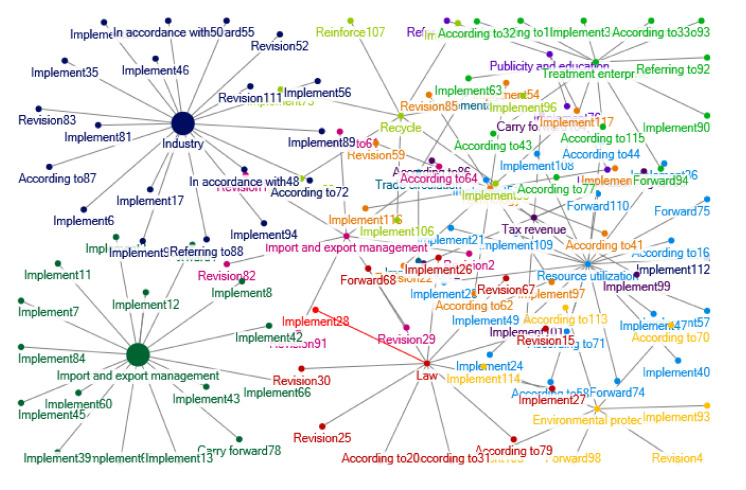
Relations of e-waste recycling policies in 2009–2012.

**Figure 12:**
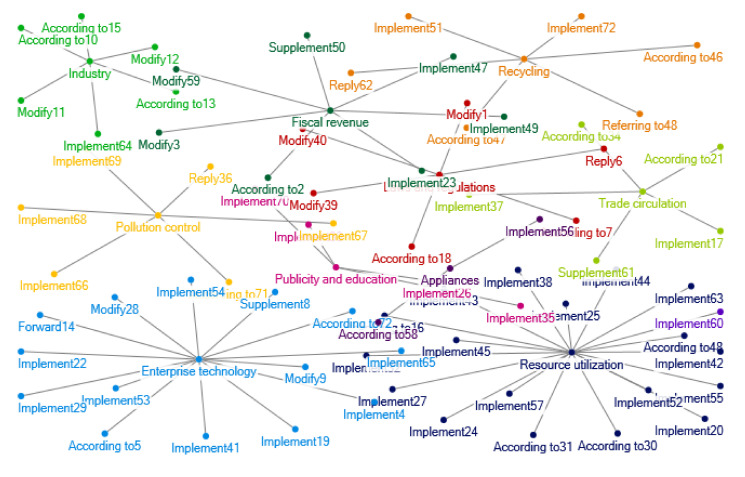
Relations of e-waste recycling policies in 2013–2016.

In Figure 9, Figure 10, Figure 11, Figure 12, the larger size of the nodes denotes larger relevant policy document quantity about the topics. In these figures, the words such as ‘Forward’, ‘Modify’, ‘Revision’, ‘Supplement’, ‘According to’, ‘Implement’ and so on, denote policies’ spread and derivation. That is: a policy from a upper government department or a same level of government department spreads to other governmental departments by ‘Forward’, ‘According to’, ‘Implement’ and so on; or a older version of a policy is improved and developed as an updated policy by ‘Modify’, ‘Revision’, ‘Supplement’ and so on to be further spread.

The policy interrelations from 2001 to 2004 are mainly related to the mineral resources in e-waste, land pollution, and protection of drinking water resources. Figure 9 shows the network of e-waste policy interrelations in 2001–2004. Usually, a main policy is a starting point of the network, and then, other policies make detailed explanation or supplement to the starting policy. For example, in 2001, the State Environmental Protection Administration issued the *“Tenth Five Year Plan” for Energy Conservation and Comprehensive Utilization of Resources*. The State Economic and Trade Commission and other departments forwarded this plan. In addition, the State Environmental Protection Administration and the Ministry of Science and Technology further improved the technical support and incentive policies for the dismantling of electrical and electronic products. In 2003, the announcement of the State Environmental Protection Administration on “*Strengthening the Environmental Management of Waste Electrical and Electronic Equipment*” was a supplementary announcement made directly in accordance with the relevant provisions of the law on the prevention and control of environmental pollution by solid waste, mainly aiming at e-waste.

In 2005–2008, there are many policies interrelations about environmental management, resource utilization, dismantling and smelting technology. Figure 10 shows the network of e-waste policy interrelations in 2005–2008. In this period, China issued some major policies such as the “*Notice of the State Council on Building a Conservation Oriented Society*”, the “*Several Opinions of the State Council on Accelerating the Development of Circular Economy*”, the “*Renewable Energy Law of the People’s Republic of China*”, and the “*Law of The People’s Republic of China on the Prevention and Control of Environmental Pollution from Solid Waste*”. Accordingly, a series of supporting policies was successively issued, such as the “*Pilot of Construction of Recycling System of Renewable Resources*”, the “*Technical Policies for Pollution Prevention and Control of Waste Household Appliances and Electronic Products*”, the “*Technical Policies for Pollution Prevention and Control of Waste Batteries*”, the “*Management Measures for Environmental Pollution Prevention and Control of Electronic Waste*”, and the “*Import and Export Management of Waste Electrical Appliances and Electronic Products*”, etc.. The policy intent of laws and regulations was gradually unfolded.

From 2009 to 2012, one of the most prominent subjects in forming policy interrelations is the old for new policy, which was introduced in May 2009, mainly for automobiles and home appliances. At the same time, this policy, together with policies of automobile going to the countryside and home appliances going to the countryside, constitute a policy system to expand the consumption of automobiles and home appliances. The policy of home appliances going to the countryside is consistent with the system design of the “*Regulations on the Administration of Recycling and Treatment of Waste Electrical and Electronic Products*” implemented in 2011, which promotes the specialized recycling of e-waste. The related policies also include promoting the circulation of used goods, which is mainly implemented by the Ministry of Commerce. The recycling and treatment of electrical and electronic products as well as the treatment qualification and technical requirements are mostly based on the “*Law of the People’s Republic of China on the Prevention and Control of Environmental Pollution by Solid Waste*” and the “*Regulations on the Management of the Recycling and Treatment of Waste Electrical and Electronic Products*”. In 2010, the general office of the Ministry of Environmental Protection, the general office of the National Development and Reform Commission, the general office of the Ministry of Industry and Information Technology, the general office of the Ministry of Commerce and other organizations jointly prepared the “*Development Plan For The Treatment of Waste Electrical and Electronic Products (2011–2015)*”, which played a guiding role for the development of this field. According to the “*Circular Economy Promotion Law of the People’s Republic of China*”, China began to build demonstration bases of urban mineral resources in 2010, regulating the dismantling and smelting of waste electrical and electronic products. In 2012, China issued the “*Opinions of the General Office of the State Council on the Establishment of a Complete and Advanced Recycling System for Waste Commodities”*. The recycling of waste commodities has become a policy hotspot, and the professional recycling of waste electrical and electronic products has also been included in this system. At this stage, the network of policies interrelations on the recycling of e-waste has been relatively mature. Figure 11 shows the network of e-waste policy interrelations in 2009–2012.

From 2013 to 2016, the policies on the recycling of e-waste are mainly based on the “*Regulations on the Recycling and Treatment of Waste Electrical and Electronic Products*”, the “*Measures for the Management of Waste Electrical and Electronic Products Processing Qualification Licensing*”, and the “*Measures for the Management of Waste Electrical and Electronic Products Processing Fund Collection and Use*”. Thus, related policies issued in this period are mainly about measures for the collection and encouragement of processing enterprise qualification, enterprise tax subsidies, imported electrical and electronic products fund, and improving the technological innovation of enterprises. Another policy hot spot is to implement the relevant provisions of the “*Circular Economy Promotion Law*”, the “*Clean Production Promotion Law*”, and the “*Regulations on The Recycling and Treatment of Waste Electrical and Electronic Products*”. Relevant policies are about pilots of EPR for electrical and electronic products so as to encourage production enterprises to carry out specialized recycling of electrical and electronic products and fulfill producer responsibility. A series of policies on energy conservation and emission reduction during the 12th Five Year Plan involve the utilization of resources. The policies on resource utilization involve the production, recovery, dismantling, treatment, smelting and other links of electrical and electronic products. Relevant government departments have carried out vigorous publicity and education work, making efforts in resource conservation, recycling, efficient utilization and so on with supplemented and improved policies. At this period, the network of policy interrelations on e-waste is more developed, especially with the introduction of the EPR policy system. Environmental protection penetrates the life cycle of electrical and electronic products. Figure 12 shows the network of e-waste policy interrelations in 2013–2016.

Based on the above analysis, the evolution of policy relations of China’s e-waste recycling policies can be summarized into the following stages: the State Council and the Ministry of Environmental Protection formulate programmatic policies, which are assigned to other functional departments for division and cooperation.

In particular, the Ministry of Environmental Protection, as the main responsible department for formulating and implementing policies, cooperates by the Ministry of Finance, the Ministry of Industry and Information Technology, the National Development and Reform Commission, the General Administration of Customs and other departments to jointly formulate and promulgate relevant policies on e-waste recycling. The process of policy-making is from general to specific. The early laws related to recycling of e-waste were the law on prevention and control of solid waste pollution, the law on promotion of cleaner production, and the law on promotion of circular economy. In 2007, the “*Special Administrative Legislation of the Administrative Measures for Pollution Control of Electronic Information Products*” was issued. In 2008, the “*Administrative Measures for Prevention and Control of Environmental Pollution from E-Waste*” was implemented. In 2011, the “*Regulations on the Management of Recycling of Waste Electrical and Electronic Products*” was implemented, and China’s specialized legislation on recycling e-waste was gradually improved.

## 5. Discussion

Governance on e-waste has evolved from being classified as solid waste governance to specifically listed as governance on waste electrical and electronic products. The evolution from general to specific shows that the recycling of e-waste has attracted the attention of more and more government departments. The evolution of policy keywords reflect that the treatment of e-waste has become increasingly professional and advanced. The government strives to build a pollution control system with scientific recycling based on the consumption habits of Chinese consumers. For example, the policy of replacing the old with the new has promoted the large-scale centralized recycling of the old household appliances. The matching dismantling subsidies policies for the old household appliances have promoted the specialized dismantling, smelting and resource reuse of the old household appliances. China draws lessons from the popular EPR system of foreign countries, conducts pilot projects in China’s large-scale home appliance enterprises, promotes the recycling of waste home appliance products. The recycling methods and governance scope have been improved increasingly towards holistic governance.

Policy collaboration among government departments is getting closer. The policy content and level are steady improved. The first stage of intergovernmental collaboration is mainly in the import and export management of e-waste, and the subsequent intergovernmental collaboration documents are mainly aimed at pollution prevention and control from e-waste. Later, the government departments jointly issued documents that mainly involve the utilization of the mineral resources contained in e-waste, proposing the “urban mineral” demonstration base, the pilot of circular economy, the recycling management of renewable resources, etc. For example, in 2011, the “*Regulations on Recycling and Treatment of Waste Electrical and Electronic Products*” was implemented, and the supporting policies of the regulations were gradually improved to ensure the smooth implementation of the regulations. The collaboration among the government departments, which is a typical character of holistic governance, promotes the improvement of the specialized recycling of e-waste.

For policy relations, the expansion of policies is a process from point to line, and then form a diffusion of policy network via the correlation between the lines. For example, in the implementation of the “*Regulations on the Management of Recycling and Treatment of Waste Electrical and Electronic Products*”, the supporting funding collection policies and the subsidy policies for recycling enterprises are correspondingly improved, and the policies such as replacing the old with the new, and the subsidy for energy-saving household appliances, are implemented. These follow-up policies contribute to support and supplement to the previous main policy.

To sum up, the development progress of China’s policy system on professional recycling of e-waste has the following characteristics. Firstly, the policy range is gradually expanded. The relevant policies gradually involve the source production of electronic and electrical products, the circulation of used electrical and electronic products, the dismantling norms of used electrical and electronic products, the subsidies of specialized dismantling enterprises, and the restrictions on the import and export of e-waste. Secondly, the effect of the policies is getting better. Especially, the governance on the manual dismantling workshops has achieved remarkable performance (for example, see http://www.mee.gov.cn/xxgk2018/xxgk/xxgk15/201908/t20190828_730337.html), and the circular economy mode is gradually launched [42,43]. Thirdly, the policy continuity is getting stronger. For example, the qualification examination of recycling enterprises, the issuance of funds, and the pilot of recycling economy industrial park, all reflect the continuity of policies. These characteristics reflect the trend towards holistic governance on e-waste recycling.

## 6. Conclusions

Governmental departments serve a guiding role in promoting e-waste recycling in China. This work demonstrates the evolution of China’s e-waste recycling policies towards holistic governance, showing how the various governmental departments have deployed guiding strategies to achieve holistic governance of e-waste recycling, contributing to answering the central question of holistic governance about how to achieve it.

The quantitative text analysis on the relevant policy documents can help to mine policy intentions and unearth governance philosophy. The methodology may also be applied to other policy progress analyses. This paper provides a new perspective for analyzing e-waste recycling policies, offering theoretical and empirical insights to holistic governance on e-waste.

Transforming and improving government functions are significant for China to launch its ambitious reform plans [44]. This work facilitates understanding the transformation and improvement of government functions on e-waste recycling. China has a unique governance structure and political environment. Therefore, the findings in this paper cannot be guaranteed to be applicable to any other context without significant comparative research and broadened theoretical framework. However, because of China’s outsize role in the global recycling market, scholars who study e-waste recycling in wealthy countries will be interested in understanding how China’s domestic recycling policy has changed in recent years.

Producers and enterprises in China need to pay attention to China’s policy progress and governance philosophy so as to respond to governmental intentions to achieve sustainable legitimacy and preferential treatment from the government [45]. With the support of network analysis and quantitative text analysis, some hidden valuable information can be mined from publicly published policy documents, contributing to corporate strategic management and decision making. So, this work may be beneficial to enlighten corporate adaptive strategies to response to governmental signals.

This work focuses on the governmental role and policy progress in promoting the professional recycling of e-waste, but whether and how the policies are effectively implemented are other issues, which needs to be further studied. Future researches are suggested to estimate of the efficiency of issued policies, and analyze why some policies succeeded exceptionally and why some policies failed; analyze contradictions in issued policies due to many involved government units with potential conflict of interest in sophisticated policy network of dependences and competences.
